# Modeling, dynamical analysis and numerical simulation of a new 3D cubic Lorenz-like system

**DOI:** 10.1038/s41598-023-33826-4

**Published:** 2023-04-24

**Authors:** Haijun Wang, Guiyao Ke, Jun Pan, Qifang Su

**Affiliations:** 1 School of Electronic and Information Engineering (School of Big Data Science), Taizhou University, Taizhou, 318000 People’s Republic of China; 2 School of Information, Zhejiang Guangsha Vocational and Technical University of construction, Dongyang, 322100 Zhejiang People’s Republic of China; 3 School of Information Engineering, GongQing Institute of Science and Technology, Gongqingcheng, 332020 People’s Republic of China; 4 Department of Big Data Science, School of Science, Zhejiang University of Science and Technology, Hangzhou, 310023 People’s Republic of China

**Keywords:** Mathematics and computing, Applied mathematics

## Abstract

Little seems to be considered about the globally exponentially asymptotical stability of parabolic type equilibria and the existence of heteroclinic orbits in the Lorenz-like system with high-order nonlinear terms. To achieve this target, by adding the nonlinear terms *yz* and $x^{2}y$ to the second equation of the system, this paper introduces the new 3D cubic Lorenz-like system: $\dot{x}=a(y - x)$, $\dot{y}=b_{1}y+b_{2}yz+b_{3}xz+b_{4}x^{2}y$, $\dot{z}= -cz + y^{2}$, which does not belong to the generalized Lorenz systems family. In addition to giving rise to generic and degenerate pitchfork bifurcation, Hopf bifurcation, hidden Lorenz-like attractors, singularly degenerate heteroclinic cycles with nearby chaotic attractors, etc., one still rigorously proves that not only the parabolic type equilibria $S_{x} = \{(x, x, \frac{x^{2}}{c})|x\in \mathbb {R}, c\ne 0\}$ are globally exponentially asymptotically stable, but also there exists a pair of symmetrical heteroclinic orbits with respect to the *z*-axis, as most other Lorenz-like systems. This study may offer new insights into revealing some other novel dynamic characteristics of the Lorenz-like system family.

## Introduction

In 1963, the introduction of the Lorenz attractor^1,2,3^ motivated scholars to reveal the forming mechanism of it and other various strange attractors^2,4–22^. Based on boundary problem and contraction map, Shilnikov et al.^23^ developed an effective tool to study the existence of homoclinic and heteroclinic orbits. When detecting homoclinic and heteroclinic trajectories of Lorenz-like systems, by aid of Lyapunov function, Leonov^24^ formulated another effective method, i.e., fishing principle, which also was applied to solve the Tricomi problem^25^. Recently, Belykh et al.^19^ pioneered a new way and developed an elegant geometrical method of synthesizing a piecewise-smooth ODE system that can switch between several linear systems with known exact solutions that can display a resembling the celebrated Lorenz attractor whose structure and bifurcations can be described rigorously without any computer assistance. Moreover, Belykh et al.^20^ performed a rigorous analysis of its homoclinic bifurcations that the emergence of sliding motions leads to novel bifurcation scenarios in which bifurcations of unstable homoclinic orbits of a saddle can yield stable limit cycles, which are in sharp contrast with their smooth analogs that can generate only unstable (saddle) dynamics. In addition, Gonchenko et al.^21,22^ studied geometrical and dynamical properties of the discrete Lorenz-like attractors and conjoined Lorenz twins in three-dimensional maps and flows. In contrast to self-excited Lorenz-like attractors, some hidden ones were coined in the Lorenz-like systems^12–14^. Meanwhile, Zhang and Chen^15^, and Kuznetsov et al.^26^ generalized the second part of the celebrated Hilbert’s 16th problem^27^ on the number and mutual disposition of attractors and repellers in the chaotic multidimensional dynamical systems, and, in particular, their dependence on the degree of polynomials in the model. From the point of view of boundedness and Lyapunov exponents, Liao et al.^28,29^ argued that the former attracts trajectories of the studied system with the way from outside to inside, and the latter pushes the trajectories with the way from inside to outside, which are two basic sufficient conditions that guarantee the studied continuous system to exhibit chaotic motions.

With the presence of powerful computational tools, scientists shifted to computer-assisted proof for the Lorenz attractor^30–32^. Dated back to 1999, based on the Lorenz system and the method of chaotification, Chen and Ueta^7^ reported the finding of a new chaotic attractor in a new system, i.e., the Chen attractor. Following this thought, many researchers later proposed many other systems which exhibit various strange attractors, the Lü attractor^8^, Li attractor^9^, Rabinovich attractor^10^, Wang-Chen attractor^11^, Sprott attractor^17^, and others^2,18–22^. Among these chaotic systems, a large number of systems^2,14,18,33–48^ related to the Lorenz system, i.e., Lorenz-like/type systems, have been intensively studied by researchers, which in turn might account for revealing the nature of the Lorenz system itself. For example, the broken version of coexisting pseudo and true singularly degenerate heteroclinic cycles, or explosion version of normally hyperbolic stable foci could create most of the Lorenz-like attractors, i.e., two-, three-, four-wing/scroll self-excited or hidden chaotic/hyperchaotic attractors^14,18,33–48^, shedding light on the forming mechanism of chaos.

In 2006, Li et al. formulated a method for proving the existence of heteroclinic orbits to the origin and two nontrivial equilibria of the Chen system, i.e., combining Lyapunov function, the definitions of both $\alpha$-limit set and $\omega$-limit set^49^. Later on, other researchers^14,37,38,42,43,47,48,50–60^ applied it to other Lorenz-like systems one after another, which thus can be considered as a general dynamical property for the Lorenz system family. However, we find that this method is not applicable to the simple Lorenz-like system (1). Fortunately, performing a similar study as the method of chaotification and introducing the nonlinear terms *yz* and $x^{2}y$ to the second equation of it, we introduce a new 3D cubic Lorenz-like system, i.e., the one (2), and present its following dynamical properties:

1. The parabolic type equilibria are globally exponentially asymptotically stable.
2. The existence of a pair of heteroclinic orbits to the origin and a pair of symmetrical equilibria.

Our study outcome not only uncovers the interesting dynamics of the cubic Lorenz-like system family, but also provides a reference on predicting the similar dynamical behaviors of other models, especially the higher dimensional ones.

Therefore, in the ongoing pursuit to determine which experimental conditions may require a more complicated model, the present work may offer characteristics of that 3D cubic Lorenz-like system which may be suitable for comparison with experimental data.

The rest of this paper is arranged as follows. Section "Preliminary" introduces some basic concepts. In Section "The new 3D cubic Lorenz-like system", one formulates a new 3D cubic Lorenz-like system and presents some basic dynamical properties of it, i.e., the Chen-like attractor and Lyapunov exponents, bifurcation analysis, singularly degenerate heteroclinic cycles or normally hyperbolic stable foci with nearby chaotic attractors. Section "Basic behaviors" studies the stability and bifurcation of equilibria by utilizing the center manifold theorem, Routh-Hurwitz criterion, the theory of pitchfork bifurcation, Hopf bifurcation and Lyapunov function. In Section "Existence of heteroclinic orbit", combining concepts of $\alpha$-limit set, $\omega$-limit set and the theory of Lyapunov function, one proves the existence of heteroclinic orbits. Conclusion remarks are drawn in Section "Conclusions".

## Preliminary

Consider the differential system $\varvec{\dot{x} = f(x, \xi )},$ where $\textbf{x}\in \mathbb {R}^{n}$ and $\mathbf {\xi } \in \mathbb {R}^{m}$ are vectors representing phase variables and control parameters respectively. Assume that $\textbf{f}$ is of class $C^{\infty }$ in $\mathbb {R}^{n}\times \mathbb {R}^{m}$. Suppose that system has an equilibrium point $\mathbf {x=x_{0}}$ at $\mathbf {\xi =\xi _{0}}$. If at least one eigenvalue of the Jacobian matrix associated with linearized vector field about $x_{0}$ is zero or has a zero real part, then $x_{0}$ is said to be non-hyperbolic or semi-hyperbolic.

In this paper, system (2) has a line of semi-hyperbolic equilibria $S_{z} = \{(0, 0, z)|z\in \mathbb {R}\}$, given by the *z*-axis. As the value of *z* varies, $S_{z}$ are saddles, or foci or nodes normally hyperbolic to the *z*-axis.

In this paper, we define the set $S_{x} = \{(x, x, \frac{x^{2}}{c})|x\in \mathbb {R}, c\ne 0\}$ to the parabolic type equilibria.

Referring to^61^, the generic pitchfork bifurcation is that the restriction of a system to the center manifold is locally topologically equivalent near the bifurcating equilibrium point to one of the following normal forms, $\dot{\xi } = m \xi \pm \xi ^{3}$. As stated in^18,47,48,51,52,62,63^, for system (2), the degenerate pitchfork bifurcation is defined to be the symmetric bifurcation occurring as the certain parameter crosses the zero value, i.e., $c = 0$, due to the line of equilibria existing for $c = 0$. The main difference between the generic and degenerate pitchfork bifurcation is that, for $c = 0$, the flow of the studied system restricted to the 1D center manifold coincides with the center manifold of the system at the origin, associated with the invariant *z*-axis, which is filled by equilibrium points if $c = 0$.

Let the set of points: *S* (either connected or disconnected) be equilibria of $\varvec{\dot{x} = f(x, \xi )}$ and $D\subset \mathbb {R}^{n}$ to be a domain containing *S*. Let $V: D\rightarrow \mathbb {R}$ be a continuously differentiable function such that $V(S)=0$ and $V(x)>0$ in $D \backslash S$, $\dot{V}(x)\le 0$ in *D*. The derivative of *V*(*x*) along the trajectories of $\varvec{\dot{x} = f(x, \xi )}$, denoted by $\dot{V}(x)$, is given by $\dot{V}(x)=\Sigma _{i=1}^{n}\frac{\partial V}{\partial x_{i}}\dot{x}_{i}=\Sigma _{i=1}^{n}\frac{\partial V}{\partial x_{i}}f_{i}(x)$. Then, *S* is stable. If $\dot{V}(x) < 0$ in $D \backslash S$, then *S* is asymptotically stable. Moreover, if $D=\mathbb {R}^{n}$, then *S* is globally asymptotically stable. In addition, $\forall \varepsilon > 0$, $V_{0}=V(t_{0})$, if $V \le V_{0}e^{-2\varepsilon (t-t_{0})}\rightarrow 0, \quad t \rightarrow + \infty$, then *S* is globally exponentially asymptotically stable.

## The new 3D cubic Lorenz-like system

Based on the Lorenz-like system^1^:

1 $$\begin{aligned} \left\{ \begin{array}{ll} \dot{x}&{}=a(y - x),\\ \dot{y}&{}=-xz+cy, a,b,c\in \mathbb {R},\\ \dot{z}&{}= -bz + y^{2}, \end{array}\right. \end{aligned}$$

one in this section proposes the following 3D autonomous chaotic system:

2 $$\begin{aligned} \left\{ \begin{array}{ll} \dot{x}&{}=a(y - x),\\ \dot{y}&{}=b_{1}y+b_{2}yz+b_{3}xz+b_{4}x^{2}y, \\ \dot{z}&{}= -cz + y^{2}, \end{array}\right. \end{aligned}$$

where $$a, c, b_{i} \in \mathbb {R}$$, $$i=1, 2, 3, 4$$.

### Remark 3.1

Referring to^1^, the results on stability and Hopf bifurcation of $P_{\pm }=(\pm \sqrt{bc},\pm \sqrt{bc},c)$ of system (1) are erroneous. To this end, we firstly derive the right result by Routh-Hurwitz criterion and Projection Method. Secondly, that system undergoes Bautin bifurcation (generalized or degenerate Hopf bifurcation) at $P_{\pm }$ when parameters *a*, *b*, *c* satisfy the golden proportion $a = \frac{1+\sqrt{5}}{2}c$, $b = \frac{-1+\sqrt{5}}{2}c$. Finally, a hidden Lorenz-like attractor coexisting with one saddle in the origin and two stable equilibria is coined based on bifurcation diagrams. The manuscript has been uploaded to the web site: “https://github.com/IjbcThree/Kim”, and the interested readers can download it.

Set $\textbf{X} = (x, y, z)^{T}$, system (2) is rewritten as

3 $$\begin{aligned} \dot{\textbf{X}}=\left( \begin{array}{ccc}-a&{}a&{}0\\ 0&{}b_{1}&{}0\\ 0&{}0&{}-c\end{array}\right) \textbf{X} +x\left( \begin{array}{ccc}0&{}0&{}0\\ 0&{}0&{}b_{3}\\ 0&{}0&{}0\end{array}\right) \textbf{X}+\left( \begin{array}{c}0\\ b_{2}yz+b_{4}x^{2}y\\ y^{2}\end{array}\right) . \end{aligned}$$

**Figure 1:**
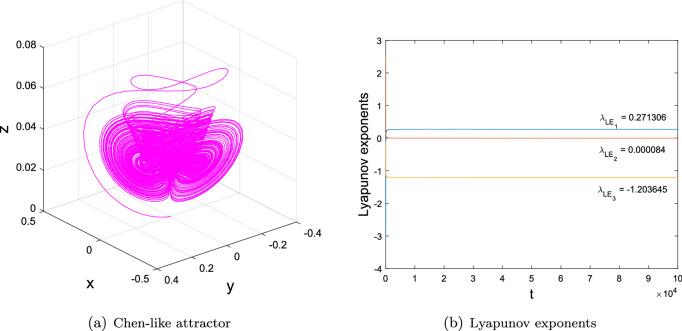
Phase portrait and Lyapunov exponents of system (2) with $(a,b_{1},b_{2},b_{3},b_{4},c) = (3,2.5,-7,-100,8,0.3)$ and $(x_{0}^{1}, y_{0}^{1}, z_{0}^{1})=(0.1382, 0.1618, 0)\times 1e^{-7}$.

Apparently, system (2) is not topologically equivalent to the generalized Lorenz systems family^2^, and it shows a Chen-like attractor with three Lyapunov exponents: $$(\lambda _{LE_{1}},\lambda _{LE_{2}},\lambda _{LE_{3}})=(0.271306,0.000084,$$ $$-1.203645)$$ when $$(a,b_{1},b_{2},b_{3},b_{4},c) = (3,2.5,-7,-100,8,0.3)$$ and $$(x_{0}^{1}, y_{0}^{1}, z_{0}^{1})=(0.1382, 0.1618, 0)\times 1e^{-7}$$, as depicted in Fig. 1.

Furthermore, the chaotic dynamics are examined in the following two cases:

(1) $$(a,b_{2},b_{3},b_{4},c) = (3,-7,-50,8,0.3)$$, $$b_{1}\in [0, 4]$$

**Figure 2:**
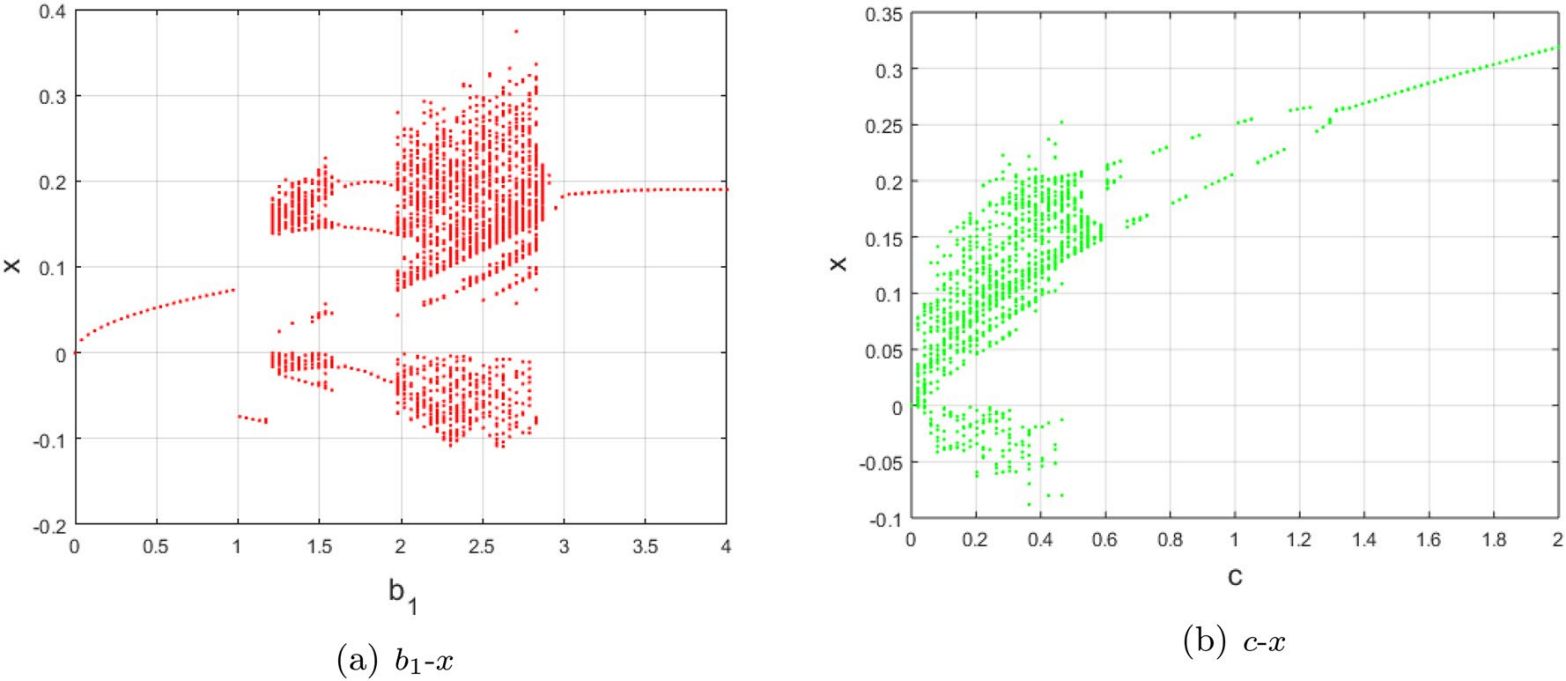
Bifurcation diagrams of system (2) with (**a**) $(a,b_{2},b_{3},b_{4},c) = (3,-7,-50,8,0.3)$, $b_{1}\in [0, 4]$ and (**b**) $(a,b_{1},b_{2},b_{3},b_{4}) = (3,2.5,-7,-100,8)$, $c\in [0, 2]$, and initial value $(x_{0}^{1}, y_{0}^{1}, z_{0}^{1})=(0.1382, 0.1618, 0)\times 1e^{-7}$.

**Figure 3:**
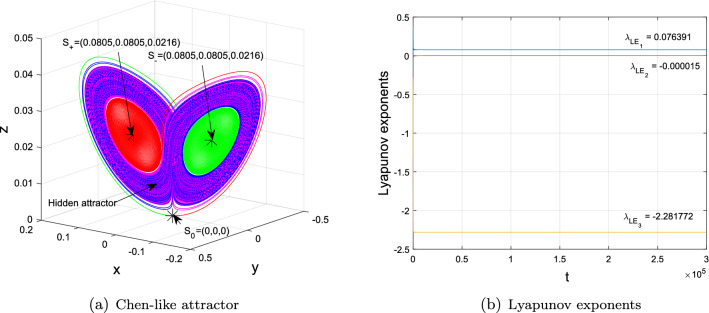
(**a**) The hidden attractor with $(a,b_{1},b_{2},b_{3},b_{4},c)=(3,1.179,-7,-50,8,0.3)$ and initial conditions $(\pm 0.05, \pm 0.03, 0.03)$, (**b**) Lyapunov exponents. Outgoing separatrices of unstable zero equilibrium $S_{0}$ with initial conditions $(x_{0}^{1,2}, y_{0}^{1,2}, z_{0}^{1})=(\pm 0.1382, \pm 0.1618, 0)\times 1e^{-7}$ tend to two symmetric stable equilibria $S_{\pm }=(\pm 0.0805,\pm 0.0805,0.0216)$.

**Figure 4:**
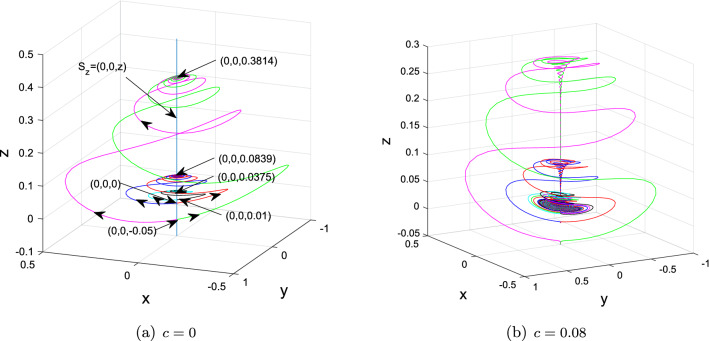
Chen-like attractor created through collapse of singularly degenerate heteroclinic cycles consisting of normally hyperbolic saddles $S_{z}^{1,2,3}$ and normally hyperbolic stable foci (0, 0, 0.3814), (0, 0, 0.0839) and (0, 0, 0.0375) when $(a,b_{1},b_{2},b_{3},b_{4},c)=(3,2.5, -7, -138,9,0)$ and $(x_{0}^{1,2}, y_{0}^{1,2})=(\pm 1.382, \pm 1.618)\times 10^{-6}$, $z_{0}^{2,1,3} = -0.05, 0, 0.01$.

In this case, based on Proposition 4.1 and 4.7 in Section "Basic behaviors", the equilibria $S_{\pm }$ exist and are stable for $b_{1}\in (0,1.2210)$. This coincides well with the bifurcation diagram in Fig. 2a. In particular, at $b_{1}=1$, trajectories of system (2) change from the stable $S_{+}$ to the stable $S_{-}$, which is a sign to chaos as the ones^14,38^, especially the hidden one illustrated in Fig. 3. Particularly, when $1.180\le b_{1}<1.2210$, there exist chaotic attractors coexisting with stable $S_{\pm }$ and the saddle $S_{0}$. While $1.2210<b_{1}<2.869$, system (2) experiences chaotic behaviors coexisting with unstable $S_{\pm }$ and the saddle $S_{0}$. But there are periodic three windows in the chaotic band for $1.576<b_{1}<1.98$. When $2.869<b_{1}<4$, there is a period-doubling bifurcation window, which is an important route to chaos and is also similar to its special case [^1^, Figure 4, p.1888].

(2) $$(a,b_{1},b_{2},b_{3},b_{4}) = (3,2.5,-7,-100,8)$$, $$c\in [0, 2]$$

**Figure 5:**
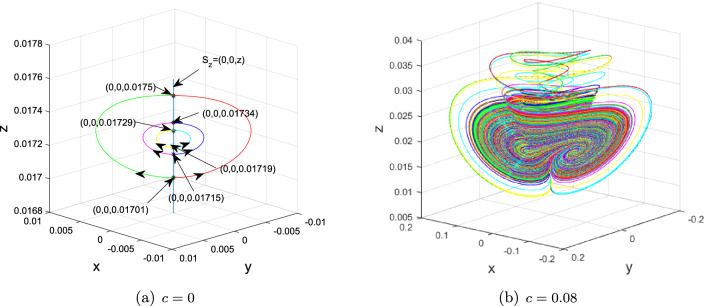
Chen-like attractor created through collapse of singularly degenerate heteroclinic cycles consisting of normally hyperbolic saddles $S_{z}^{4,5,6}$ and normally hyperbolic stable nodes (0, 0, 0.0175), (0, 0, 0.01734) and (0, 0, 0.01729) when $(a,b_{1},b_{2},b_{3},b_{4},c)=(3,2.5, -7, -138,9,0)$ and $(x_{0}^{1,2}, y_{0}^{1,2})=(\pm 1.382, \pm 1.618)\times 10^{-6}$, $z_{0}^{4,5,6} = 0.01701,0.01715,0.01719$.

At this time, from Proposition 4.1, 4.2 and 4.7 in Sectipn "Basic behaviors", the non-isolated or line of semi-hyperbolic equilibria $S_{z}$ exist for $c=0$, and the equilibria $S_{\pm }$ also exist and are stable for $c\in (3.0234,10.8865)$. For $c=0$, singularly degenerate heteroclinic cycles and normally hyperbolic stable foci $S_{z}$ with nearby chaotic attractors exist, as shown in Fig. 2b, which is in accordance with Figs. 4 and 5, despite a little bit on the parameters $b_{3}$ and $b_{4}$. When $0<c<0.6$, system (2) undergoes chaotic behaviors coexisting with unstable $S_{\pm }$ and the saddle $S_{0}$. While $0.6<c<0.2$, there is a period-doubling bifurcation window, foreboding a coming chaos.

### Remark 3.2

In contrast with other Lorenz-like systems^12–14,51^ and system (1) i.e., a special case of system (2), it is a difficult task to detect the hidden Lorenz-like attractors in system (2), which might contribute to the power of nonlinear terms and the number of parameters.

Further, based on the dynamics of $S_{z}$ in Proposition 4.4 in Section "Existence of heteroclinic orbit" and through a detailed numerical study, we may state the following numerical result.

$\mathbf {Numerical\quad Result \,3.1}$ If $c=0$ and $a[b_{1}+(b_{2}+b_{3})z_{1}] > 0$ for $z_{1}\in \mathbb {R}$, then the 1D unstable manifolds $W^{u}(S_{z}^{1})$ ($S_{z}^{1}=(0, 0, z_{1})$) of each normally hyperbolic saddle $S_{z}^{1}$ given in Proposition 4.4 tend to one of the normally hyperbolic stable nodes (resp. foci) $S_{z}^{2}=(0, 0, z_{2})$ as $t\rightarrow \infty$, where $z_{2}$ satisfies $b_{1}+b_{2}z_{2}-a < 0$, $a[b_{1}+(b_{2}+b_{3})z_{2}] < 0$ and $(\tau _{1})^{2} + 4\rho _{1} = (a-b_{1}-b_{2}z)^{2} + 4a[b_{1}+(b_{2}+b_{3})z] \ge 0$ (resp. $<0$), which together with the line of equilibria between $S_{z}^{1}$ and $S_{z}^{2}$ forms singularly degenerate heteroclinic cycles. With a small perturbation of $c>0$, the broken version of singularly degenerate heteroclinic cycles, or explosions of normally hyperbolic stable nodes or foci creates chaotic attractors.

Take, for instance, $(a,b_{1},b_{2},b_{3},b_{4},c)=(3,2.5, -7, -138,9,0)$ and $(x_{0}^{1,2}, y_{0}^{1,2})=(\pm 1.382, \pm 1.618)\times 10^{-6}$, $z_{0}^{2,1,3,4,5,6,7,8,9} = -0.05, 0, 0.01,0.01701,0.01715,0.01719, 0.0173, 0.02, 0.035$. At this time, the dynamics of $S_{z}$ are included in Table 1 when the value of *z* varies.

**Figure 6:**
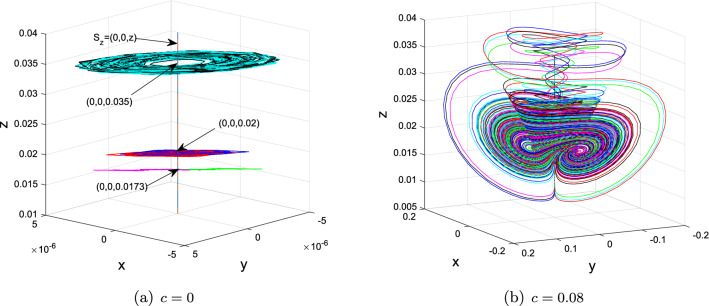
Chen-like attractor created through explosions of normally hyperbolic stable foci when $(a,b_{1},b_{2},b_{3},b_{4})=(3,2.5, -7, -138,9)$ $(x_{0}^{1,2}, y_{0}^{1,2})=(\pm 1.382, \pm 1.618)\times 10^{-6}$, $z_{0}^{7,8,9}=0.0173, 0.02, 0.035$.

**Figure 7:**
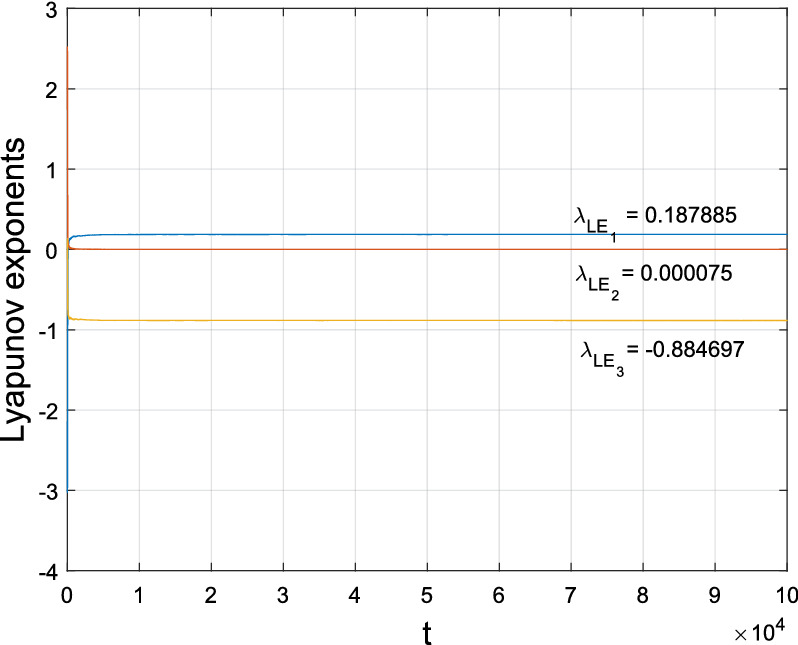
Lyapunov exponents of the bifurcated Chen-like attractor when $(a,b_{1},b_{2},b_{3},b_{4},c)=(3,2.5, -7, -138,9,0.08)$.

The 1D $W^{u}(S_{z}^{1,2,3})$ (resp. $W^{u}(S_{z}^{4,5,6})$) of normally hyperbolic saddles $S_{z}^{2,1,3}=$ $(0,0,-0.05)$, (0, 0, 0), (0, 0, 0.01) (resp. $S_{z}^{4,5,6}=$ (0, 0, 0.01701), (0, 0, 0.01715), (0, 0, 0.01719)) tend upward the normally hyperbolic stable foci (0, 0, 0.3814), (0, 0, 0.0839) and (0, 0, 0.0375) (resp. nodes (0, 0, 0.0175), (0, 0, 0.01734) and (0, 0, 0.01729)) in $S_{z}$ as $t\rightarrow \infty$, forming singularly degenerate heteroclinic cycles, which further also collapse into Chen-like attractor depicted in Figs. 4, 5 and 7 when $c = 0.08$. Moreover, as shown in Fig. 6, explosions of normally hyperbolic stable foci (0, 0, 0.0173), (0, 0, 0.02) and (0, 0, 0.035) also create Chen-like attractors. Figures 4, 5 and 6 only depict some of them. The existence of infinitely many $S_{z}$ given in Proposition 4.3 suggests that there exists an infinite set of singularly degenerate heteroclinic cycles and normally hyperbolic stable nodes and foci.

### Remark 3.3

Set $$(a,b_{1},b_{2},b_{3},b_{4},c)=(3,2.5, -7, -138,9,0)$$.

**Figure 8:**
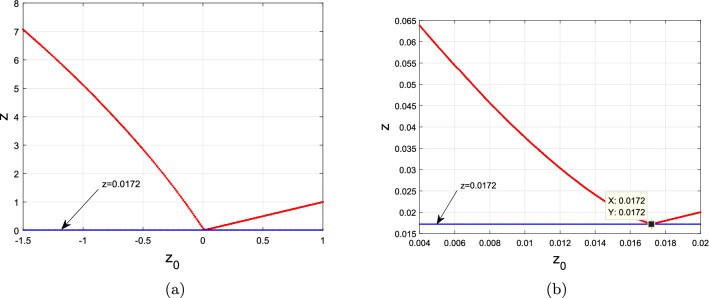
Bifurcation diagrams of the system (2) with $(a,b_{1},b_{2},b_{3},b_{4},c)=(3,2.5, -7, -138,9,0)$ and $(x_{0}^{1}, y_{0}^{1})=(1.382, 1.618)\times 10^{-6}$ and (**a**) $z_{0}\in [-1.5, 1]$, (**b**) $z_{0}\in [0.004,0.02]$.

(1) If $z < 0.01701$, the trajectories of system (2) starting from the unstable manifold of $S_{z} = (0, 0, z)$ ultimately toward the normally hyperbolic stable foci $S_{z}$ with $z > 0.0175$, forming singularly degenerate heteroclinic cycles, as depicted in Figs. 4a and 8.

(2) If $0.01701 \le z < 0.0172$, the trajectories of system (2) starting from the unstable manifold of $S_{z} = (0, 0, z)$ ultimately toward the normally hyperbolic stable nodes $S_{z}$ with $0.0172 < z \le 0.0175$, forming singularly degenerate heteroclinic cycles, as depicted in Figs. 5a and 8.

(3) If $z > 0.0172$, all of $S_{z} = (0, 0, z)$ are normally hyperbolic stable nodes or node-foci, as shown in Figs. 6a and 8.

**Table 1:** The dynamics of $S_{z}$ with $(a,b_{1},b_{2},b_{3},b_{4},c)=(3,2.5, -7, -138,9,0)$ and $z \in \mathbb {R}$.

*z*	$[35.3499, \infty )$	(0.0175, 35.3499)	(0.0172, 0.0175]	0.0172	$(-\infty , 0.0172)$
$S_{z}$	Stable node	Stable focus	Stable node	a 1D $W_{loc}^{s}$ and a 2D $W_{loc}^{c}$	Saddle

## Basic behaviors

In this section, the stability and bifurcation of equilibria of system (2) are studied by aid of the center manifold theorem, Routh-Hurwitz criterion, the theory of pitchfork bifurcation and Hopf bifurcation, Lyapunov function and so on.

Firstly, from the algebraic structure of system (2), one easily presents the distribution of equilibrium points in the following proposition.

### Proposition 4.1

(i) When $c = 0$, $S_{z} = \{(0, 0, z)|z\in \mathbb {R}\}$ is a line of semi-hyperbolic equilibria of system (2).

(ii) When $b_{1} = 0$, $c \ne 0$ and $cb_{4}+b_{3}+b_{2}=0$, $S_{x} = \{(x, x, \frac{x^{2}}{c})|x\in \mathbb {R}, c\ne 0\}$ is the parabolic type equilibria.

(iii) While $cb_{1}[cb_{4}+b_{3}+b_{2}] < 0$, system (2) has three equilibria: $S_{0} = (0, 0, 0)$ and a pair of symmetrical equilibria

$$\begin{aligned} S_{\pm } = (\pm \sqrt{-\frac{cb_{1}}{cb_{4}+b_{3}+b_{2}}}, \pm \sqrt{-\frac{cb_{1}}{cb_{4}+b_{3}+b_{2}}}, -\frac{b_{1}}{cb_{4}+b_{3}+b_{2}}). \end{aligned}$$

Secondly, for the convenience of determining the stability and bifurcation of equilibria, one has to calculate Jacobian matrix associated vector field of system (2):

$$\begin{aligned} J=\left( \begin{array}{lll} -a &{} a &{}0 \\[3pt] b_{3}z+2b_{4}xy &{}b_{1}+b_{2}z+b_{4}x^{2} &{} b_{2}y+b_{3}x \\[3pt] 0 &{}2y &{} -c \end{array}\right) .\end{aligned}$$

One can easily calculate the characteristic equations of points of $S_{z}$, $S_{0}$, $S_{x}$ and $S_{\pm }$:

1. The one of each of $S_{z}$ is
  $$\begin{aligned} \lambda [\lambda ^{2}-(b_{1}+b_{2}z-a)\lambda -a(b_{1}+(b_{2}+b_{3})z)]=0 \end{aligned}$$
  with $$\lambda _{1}=0$$, $$\lambda _{2,3} = \frac{(b_{1}+b_{2}z-a)\pm \sqrt{(b_{1}+b_{2}z-a)^{2}+4a(b_{1}+(b_{2}+b_{3})z)}}{2}$$.

(2) The one of $S_{0}$ is
  $$\begin{aligned} (\lambda +a)(\lambda -b_{1})(\lambda +c)=0 \end{aligned}$$
  with $$\lambda _{1}=-a$$, $$\lambda _{2} = b_{1}$$ and $$\lambda _{3}=-c$$.

(3) For $b_{1} = 0$, $c \ne 0$ and $cb_{4}+b_{3}+b_{2}=0$, the one of each of $S_{x}$ is
  $$\begin{aligned} \lambda [\lambda ^{2}+(a+c+\frac{b_{3}x^{2}}{c})\lambda +ac+x^{2}(b_{3}+2cb_{4})]=0 \end{aligned}$$
  with $$\lambda _{1}=0$$, $$\lambda _{2,3} = \frac{-(a+c+\frac{b_{3}x^{2}}{c})\pm \sqrt{(a+c+\frac{b_{3}x^{2}}{c})^{2}-4x^{2}(ac+b_{3}+2cb_{4})}}{2}$$.

(4) The one of $S_{\pm }$ is:
  4 $$\begin{aligned} \begin{array}{l} \lambda ^{3}+(a+c-\frac{b_{1}b_{3}}{cb_{4}+b_{3}+b_{2}})\lambda ^{2}+c(a+\frac{b_{1}(2b_{2}+b_{3}+2ab_{4})}{cb_{4}+b_{3}+b_{2}})\lambda +2acb_{1}=0. \end{array} \end{aligned}$$

**Table 2:** The local dynamical behaviors of points of $S_{z}$.

$\rho _{1}$	$\tau _{1}$	$\sigma _{1}$	Property of $S_{z}$
$$<0$$	$$ < 0$$	$$ < 0$$	Stable foci normally hyperbolic to $$S_{z}$$
	$$ < 0$$	$$ \ge 0$$	Stable nodes normally hyperbolic to $$S_{z}$$
	$$ = 0$$	$$ < 0$$	Fold-Hopf bifurcation may occur
	$$ > 0$$	$$ < 0$$	Unstable foci normally hyperbolic to $$S_{z}$$
	$$ > 0$$	$$ \ge 0$$	Unstable nodes normally hyperbolic to $$S_{z}$$
	$$ < 0$$		A 1D $$W_{loc}^{s}$$ and a 2D $$W_{loc}^{c}$$
$$=0$$	$$ = 0$$		A 3D $$W_{loc}^{c}$$
	$$ > 0$$		A 2D $$W_{loc}^{c}$$ and a 1D $$W_{loc}^{u}$$
$>0$			Saddles normally hyperbolic to $S_{z}$

**Table 3:** The dynamical behaviors of points of $S_{x}$.

$\rho _{2}$	$\tau _{2}$	$\sigma _{2}$	Property of $S_{x}$
$$>0$$	$$ < 0$$	$$ < 0$$	Stable foci normally hyperbolic to $$S_{x}$$
	$$ < 0$$	$$ \ge 0$$	Stable nodes normally hyperbolic to $$S_{x}$$
	$$ = 0$$	$$ < 0$$	Fold-Hopf bifurcation may occur
	$$ > 0$$	$$ < 0$$	Unstable foci normally hyperbolic to $$S_{x}$$
	$$ > 0$$	$$ \ge 0$$	Unstable nodes normally hyperbolic to $$S_{x}$$
	$$ < 0$$		A 1D $$W_{loc}^{s}$$ and a 2D $$W_{loc}^{c}$$
$$=0$$	$$ = 0$$		A 3D $$W_{loc}^{c}$$
	$$ > 0$$		A 2D $$W_{loc}^{c}$$ and a 1D $$W_{loc}^{u}$$
$<0$			Saddles normally hyperbolic to $S_{x}$

### Proposition 4.2

(1) A generic pitchfork bifurcation happens at $S_{0}$ when $b_{1}$ crosses the null value and $cb_{1}(cb_{4}+b_{3}+b_{2}) < 0$. (2) If *c* passes through the null value and $cb_{1}(cb_{4}+b_{3}+b_{2}) < 0$, then system (2) undergoes a degenerate pitchfork bifurcation at $S_{z}$.

### Proof

(1) Assume $c(cb_{4}+b_{3}+b_{2}) \ne 0$, $c \ne 0$ and $b_{1} = 0$. The matrix associated with the vector field (2) linearized about $S_{0}$ has the eigenvalues: $\lambda _{1} = -a$, $\lambda _{2} = b_{1}=0$ and $\lambda _{3} = -c$ with the corresponding eigenvectors

$$ (\xi _{1} ,\;\xi _{2} ,\;\xi _{3} ) = \left( {\begin{array}{*{20}c}    1 & 1 & 0  \\    0 & 1 & 0  \\    0 & 0 & 1  \\   \end{array} } \right). $$

Set $\bar{b}_{1} = b_{1} - 0$. System (2) becomes

5 $$\begin{aligned} \left\{ \begin{array}{ll} \dot{x}&{}=a(y - x),\\ \dot{y}&{}=\bar{b}_{1} y+b_{2}yz+b_{3}xz+b_{4}x^{2}y, \\ \dot{z}&{}= -cz + y^{2}. \end{array}\right. \end{aligned}$$

Next, the following transformation

$$\begin{aligned} (x, y, z)^{T} = (\xi _{2}, \xi _{1}, \xi _{3})(u, v, s)^{T}, \end{aligned}$$

converts system (5) into

6 $$\begin{aligned} \left( \begin{array}{l} \dot{u}\\ \dot{v}\\ \dot{s}\end{array}\right) =\left( \begin{array}{lll}0&{}0&{}0\\ 0&{}-a&{}0\\ 0&{}0&{}-c\end{array}\right) \left( \begin{array}{l}u\\ v\\ s\end{array}\right) +\left( \begin{array}{l}\bar{b}_{1}u+b_{2}us+b_{3}(u+v)s+b_{4}(u+v)^{2}u\\ -(\bar{b}_{1}u+b_{2}us+b_{3}(u+v)s+b_{4}(u+v)^{2}u)\\ -cs+u^{2} \end{array}\right) . \end{aligned}$$

Based on the center manifold theorem, one can study the two-parameter family of first-order ordinary differential equations on the center manifold of $S_{0}$:

$$ \begin{aligned}   W_{{loc}}^{c} (S_{0} ) =  & \{ (u,\;\bar{b}_{1} ,\;v,\;s) \in \mathbb{R}^{4} |v = V(u,\;\bar{b}_{1} ),s = S(u,\;\bar{b}_{1} ), \\     & V(0,\;0) = S(0,\;0) = 0,\;DV(0,\;0) = DS(0,\;0) = 0\}  \\  \end{aligned}    $$

to determine the stability of $S_{0}$ near $\bar{b}_{1} = 0$.

Therefore, one arrives at

7 $$ \begin{array}{*{20}l}    {V(u,\;\bar{b}_{1} ) =  - \frac{{b_{2}  + cb_{4} }}{{ac}}u^{3}  + O(\parallel (u,\;\bar{b}_{1} )\parallel ^{3} ),} \hfill  \\    {S(u,\;\bar{b}_{1} ) = \frac{1}{c}u^{2}  + O(\parallel (u,\;\bar{b}_{1} )\parallel ^{3} ),} \hfill  \\   \end{array}  $$

through substituting the expanded expressions of $V(u, \bar{b}_{1})$ and $S(u, \bar{b}_{1})$:

$$ \begin{array}{*{20}l}    {V(u,\;\overline{{b_{1} }} ) = \Sigma _{{i + j = 2}}^{\infty } v_{{ij}} u^{i} \overline{{b_{1} }} ^{j} ,} \hfill  \\    {S(u,\;\overline{{b_{1} }} ) = \Sigma _{{i + j = 2}}^{\infty } s_{{ij}} u^{i} \overline{{b_{1} }} ^{j} ,} \hfill  \\   \end{array}  $$

into system (6).

Further, the restricted vector field of system (6) on its center manifold

8 $$ \left\{ {\begin{array}{*{20}l}    {\dot{u}} \hfill & { = \bar{b}_{1} u + \frac{{cb_{4}  + b_{3}  + b_{2} }}{c}u^{3}  + O(\parallel (u,\;\bar{b}_{1} )\parallel ^{4} )\mathop  = \limits^{\Delta } U(u,\;\bar{b}_{1} ),} \hfill  \\    {\dot{\bar{b}}_{1} } \hfill & { = 0,} \hfill  \\   \end{array} } \right. $$

is obtained by substituting those expressions in (7) into system (6).

Since $$U(0, 0) = 0, \quad \frac{\partial {U}}{\partial {u}}\big |_{u=0, \bar{b}_{1}=0} = 0$$ and

$$ \left\{ {\begin{array}{*{20}l}    {\frac{{\partial U}}{{\partial \bar{b}_{1} }}|_{{u = 0,\bar{b}_{1}  = 0}} } \hfill & { = 0,} \hfill  \\    {\frac{{\partial ^{2} U}}{{\partial u^{2} }}|_{{u = 0,\bar{b}_{1}  = 0}} } \hfill & { = 0,} \hfill  \\    {\frac{{\partial ^{2} U}}{{\partial u\partial \bar{b}_{1} }}|_{{u = 0,\bar{b}_{1}  = 0}} } \hfill & { = 1,} \hfill  \\    {\frac{{\partial ^{3} U}}{{\partial u^{3} }}|_{{u = 0,\bar{b}_{1}  = 0}} } \hfill & { = \frac{{cb_{4}  + b_{3}  + b_{2} }}{c} \ne 0,} \hfill  \\   \end{array} } \right. $$

a generic pitchfork bifurcation happens at $S_{0}$ according to the pitchfork bifurcation theory^23,64–66^.

(2) When the parameter *c* crosses the zero value, the family of this vector field crosses this degenerate situation transversally. More precisely speaking, for $cb_{1}[cb_{4}+b_{3}+b_{2}] < 0$, the line of equilibria $S_{z}$ existing for $c=0$ disappears and equilibria $S_{0}$ and $S_{\pm }$ appear in system (2).

The proof is over. $\square$

### Proposition 4.3

Assume $a > 0$, $b_{1} > 0$ and $c > 0$. The saddle $S_{0}$ has a 1D $W_{loc}^{u}(S_{0})$ that is locally characterized by

9 $$\begin{aligned} W^{u}_{loc}(S_{0})=\left\{ \left( \begin{array}{l}x\\ y\\ z\end{array}\right) \Bigg | \begin{array}{lll} |x|\ll 1,\\ y=\frac{a+b_{1}}{a}x+O(x^2),\\ z=\frac{(a+b_{1})^{2}}{2b_{1}a^{2}}x^2+O(x^3), \end{array} \right\} \end{aligned}$$

and a 2D $W_{loc}^{s}(S_{0})$ containing the *z*-axis.

### Proof

The proof is similar to the ones in^37,52,54,56^. One only sketches it. For $a > 0$, $b_{1} > 0$ and $c > 0$, the eigenvalues of $S_{0}$ are $\lambda _{1} = -a < 0$, $\lambda _{2} = b_{1}>0$ and $\lambda _{3} = -c<0$. Thus $S_{0}$ has a 2D $W_{loc}^{s}$ containing *z*–axis, and 1D $W^{u}_{loc}(S_{0})$ whose appropriate expression is

$$\begin{aligned} W^{u}_{loc}(S_{0})=\left\{ \left( \begin{array}{c}x\\ y\\ z\end{array}\right) \Bigg |\begin{array}{c}|x|\ll 1,\\ y=H(x),\\ z=K(x), \end{array}\begin{array}{c} A_{1}\left( \begin{array}{c}1\\ H'(0)\end{array}\right) =\lambda _{2}\left( \begin{array}{c}1\\ H'(0)\end{array} \right) ,\\ H(0)=K(0)=0,\end{array}\right\} \end{aligned}$$

with $$A_{1}=\left( \begin{array}{cc} -a &{} a\\ 0&{} b_{1} \end{array}\right) . $$ Assuming that $y=H(x)=H_{1}x+H_{2}x^2+O(x^3)$ and $z=K(x)=K_{1}x+K_{2}x^2+O(x^3)$, and substituting them into system (2), one obtains the following first-order differential equation $K'(x)[a(H(x)-x)] = -cK(x)+(H(x))^{2},$ and

10 $$\begin{aligned} \left\{ \begin{array}{ll} b_{1}H_{1}=H_{1}a(H_{1}-1),\\ b_{1}H_{2}+b_{2}K_{1}H_{1}+b_{3}K_{1}=2H_{2}a(H_{1}-1)+H_{1}H_{2}a,\\ cK_{1}=K_{1}a(H_{1}-1),\\ H_{1}^{2}=2K_{2}a(H_{1}-1)+aK_{1}H_{2}. \end{array}\right. \end{aligned}$$

In addition, the matrix equation $$\left( \begin{array}{cc}-a&{}a\\ 0&{}b_{1}\end{array}\right) \left( \begin{array}{c}1\\ H'(0)\end{array}\right) =\lambda _{2}\left( \begin{array}{c}1\\ H'(0)\end{array}\right) $$ suggests $H_{1}=H'(0)=\frac{a+b_{1}}{a}$. Hence, from Eq. (10), one has $K_{1}=0$, $H_{2}=0$ and $K_{2} = \frac{(a+b_{1})^{2}}{2b_{1}a^{2}}$. The proposition is thus proved. $\square$

### Proposition 4.4

(1) When $c=0$, $a,b_{1},b_{2},b_{3},b_{4}, z\in \mathbb {R}$, the local dynamical behaviors of $S_{z}$ are totally summarized in Table 2, where $\rho _{1} = a[b_{1}+(b_{2}+b_{3})z]$, $\tau _{1} = -(a-b_{1}-b_{2}z)$, and $\sigma _{1} = (\tau _{1})^{2} + 4\rho _{1} = (a-b_{1}-b_{2}z)^{2} + 4a[b_{1}+(b_{2}+b_{3})z]$. While $b_{1} = 0$, $c \ne 0$, $cb_{4}+b_{3}+b_{2}=0$ and $z\in \mathbb {R}$, Table 3 lists the local dynamics of $S_{x}$, where $\rho _{2} = ac+x^{2}(b_{3}+2cb_{4})$, $\tau _{2} = -[a+c+\frac{b_{3}x^{2}}{c}]$, and $\sigma _{2} = (\tau _{2})^{2} - 4\rho _{2} = (a+c+\frac{b_{3}x^{2}}{c})^{2} - 4[ac+x^{2}(b_{3}+2cb_{4})]$.

(2) Moreover, for $c = 2a > 0$, $b_{1} = b_{3} = 0$ and $b_{2} = -cb_{4} < 0$, each point of $S_{x}$ is globally exponentially asymptotically stable.

### Proof

(1) Firstly, the local stability of points of $S_{z}$ and $S_{x}$ easily follows from the linear analysis and is omitted here.

(2) Secondly, we discuss the global stability of points of $S_{x}$, i.e., each point of $S_{x}$ is globally exponentially asymptotically stable. For $c = 2a > 0$, $b_{1} = b_{3} = 0$ and $b_{2} = -cb_{4} < 0$, set the following Lyapunov function:

$$\begin{aligned} U=\frac{1}{2}[2a(y-x)^{2}+b_{4}(-cz+x^{2})^{2}] \end{aligned}$$

with

11 $$\begin{aligned} \begin{array}{lll} \frac{dU}{dt}\big |_{(2)}&{}=&{}-2a^{2}(y-x)^{2}-2ab_{4}(-cz+x^{2})^{2} \\ &{}=&{}-2a[a(y-x)^{2}+b_{4}(-cz+x^{2})^{2}] \\ &{}=&{}-2a U - ab_{4}(-cz+x^{2})^{2} \\ &{}\le &{}-2a U, \end{array} \end{aligned}$$

which yields

12 $$\begin{aligned} 0 \le U \le U_{0}e^{-2a(t-t_{0})}\rightarrow 0, \quad t \rightarrow + \infty . \end{aligned}$$

Namely, points of $S_{x}$ are globally exponentially asymptotically stable. The proof is finished. $\square$

### Remark 4.5

In contrast with other Lorenz-like systems^14,37,38,42,43,47–60^ and the special case of system (2), it follows from Proposition 4.4 that system (1) has no globally exponentially asymptotically stable parabolic type equilibria.

### Proposition 4.6

(1) If $a<0$ or $b_{1}>0$ or $c<0$, then $S_{0}$ is unstable. If $a>0$, $b_{1}<0$ and $c>0$, then $S_{0}$ is stable.

(2) If $c=0$, then the dynamics of $S_{0}$ are the same to the ones of $S_{z}$ with $z=0$ and listed in Table 2.

### Proof

The local stability of $S_{0}$ easily follows from the linear analysis and is omitted here. $\square$

### Proposition 4.7

Denote $$W=\{(a,b_{1},b_{2},b_{3},b_{4},c)\in \mathbb {R}^{6}|a>0, cb_{1}[cb_{4}+b_{3}+b_{2}] < 0\}$$, $$W_{2}=W\backslash W_{1}$$, $$ W_{1}=\{(a,b_{1},b_{2},b_{3},b_{4},c)\in W: a+c-\frac{b_{1}b_{3}}{cb_{4}+b_{3}+b_{2}}>0, c(a+\frac{b_{1}(2b_{2}+b_{3}+2ab_{4})}{cb_{4}+b_{3}+b_{2}})>0, 2acb_{1}>0\} $$.

$$ \begin{array}{*{20}l}    {W_{1}^{1}  = \{ (a,\;b_{1} ,\;b_{2} ,\;b_{3} ,\;b_{4} ,\;c) \in W_{1} :} \hfill & {\Gamma  < 0\} ,} \hfill  \\    {W_{1}^{2}  = \{ (a,\;b_{1} ,\;b_{2} ,\;b_{3} ,\;b_{4} ,\;c) \in W_{1} :} \hfill & {\Gamma  = 0\} } \hfill  \\    {W_{1}^{3}  = \{ (a,\;b_{1} ,\;b_{2} ,\;b_{3} ,\;b_{4} ,\;c) \in W_{1} :} \hfill & {\Gamma  > 0\} ,} \hfill  \\   \end{array}  $$

where $\Gamma = c[(a+c-\frac{b_{1}b_{3}}{cb_{4}+b_{3}+b_{2}})(a+\frac{b_{1}(2b_{2}+b_{3}+2ab_{4})}{cb_{4}+b_{3}+b_{2}})-2ab_{1}]$. $S_{\pm }$ are unstable when $(a,b_{1},b_{2},b_{3},b_{4},c)\in W_{1}^{1}\cup W_{2}$ whereas $S_{\pm }$ are asymptotically stable when $(a,b_{1},b_{2},b_{3},b_{4},c)\in W_{1}^{3}$. When $(a,b_{1},b_{2},b_{3},b_{4},c)\in W_{1}^{2}$, system (2) undergoes Hopf bifurcation at $S_{\pm }$ respectively.

### Proof

According to Routh-Hurwitz criterion and Eq. (4), $S_{\pm }$ are unstable when $(a,b_{1},b_{2},b_{3},b_{4},c)\in W_{1}^{1}\cup W_{2}$ whereas $S_{\pm }$ are asymptotically stable when $(a,b_{1},b_{2},b_{3},b_{4},c)\in W_{1}^{3}$.

While $(a,b_{1},b_{2},b_{3},b_{4},c)\in W_{1}^{2}$, Eq. (4) has one negative real root $\lambda _{1} = -(a+c-\frac{b_{1}^{*}b_{3}}{cb_{4}+b_{3}+b_{2}}) < 0$ and a pair of conjugate purely imaginary roots $\lambda _{2, 3}=\pm \omega i$, where $\omega =\sqrt{c(a+\frac{b_{1}^{*}(2b_{2}+b_{3}+2ab_{4})}{cb_{4}+b_{3}+b_{2}})}$ and $b_{1}^{*}$ satisfies $(a+c-\frac{b_{1}^{*}b_{3}}{cb_{4}+b_{3}+b_{2}})(a+\frac{b_{1}^{*}(2b_{2}+b_{3}+2ab_{4})}{cb_{4}+b_{3}+b_{2}})-2ab_{1}^{*}=0$. Then calculating the derivatives on both sides of Eq. (4) with respect to the parameter $b_{1}$ and substituting $\lambda _{2}$ and $b_{1}$ with $\omega i$ and $b_{1}^{*}$ into the derivative yield

$$\begin{aligned} \frac{d\textit{Re}(\lambda _{2})}{db_{1}}\bigg |_{b_{1}=b_{1}^{*}}=\frac{2ac(cb_{4}+b_{3}+b_{2})+b_{3}\omega ^{2}+c\lambda _{1}(2b_{2}+b_{3}+2ab_{4})}{2(\omega ^{2}+\lambda _{1}^{2})(cb_{4}+b_{3}+b_{2})} \ne 0. \end{aligned}$$

**Figure 9:**
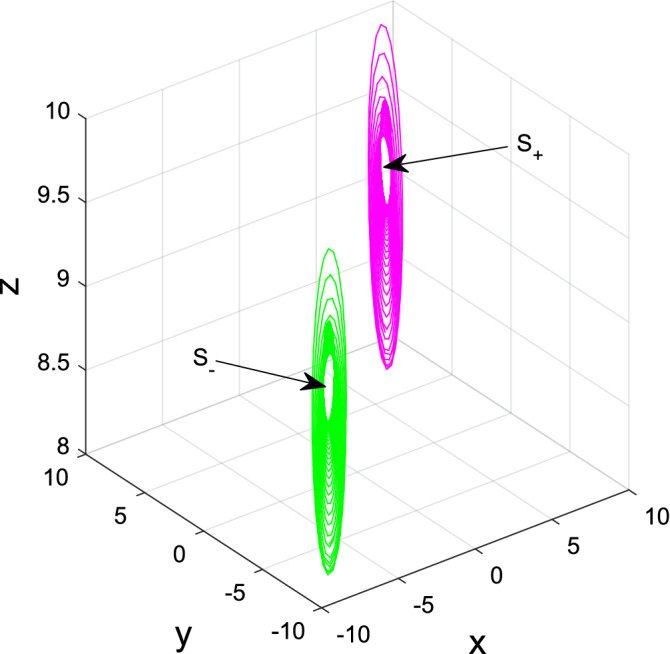
Hopf bifurcations at $S_{\pm }$ for system (2) when $(a,b_{1},b_{2},b_{3},b_{4},c)=(3,9.3563, -7, -1,1,7)$ and $(x_{0}^{3,4}, y_{0}^{3,4}, z_{0}^{10})=(\pm 7.8, \pm 7.8, 8.2)$.

Hence, the transversal condition holds. So, the Hopf bifurcations happen at $S_{\pm }$. Figure 9 illustrates that the numerical simulation agrees with the theoretical analysis. The proof is finished. $\square$

In the following Section "Conclusions", one studies the existence of heteroclinic orbits of system (2). For the convenience of discussion in the sequel, the following notations are introduced.

Denote by $\phi _{t}(q_{0}) = (x(t; x_{0}), y(t; y_{0}), z(t; z_{0}))$ a solution of system (2) with the initial condition $q_{0} = (x_{0}, y_{0}, z_{0})$. Let $\gamma ^{+}$ (resp. $\gamma ^{-}$) be the positive (resp. negative) branch of the unstable manifold $W^{u}(S_{0})$ corresponding to $x_{+} > 0$ (resp. $x_{+} < 0$) for large negative *t*, i.e., $\gamma ^{\pm } =\{\phi _{t}^{\pm }(q_{0})|\phi _{t}^{\pm }(q_{0})=(\pm x_{+}(t; x_{0}), \pm y_{+}(t; y_{0}), z_{+}(t; z_{0}))\in W_{\pm }^{u}, t\in \mathbb {R}\}$.

## Existence of heteroclinic orbit

Combining the Lyapunov function, concepts of both $\alpha$-limit set and $\omega$-limit set^14,37,38,42,43,47–60^, one in this section rigorously proves the existence of a pair of heteroclinic orbits of system (2). Firstly, the unstable manifold of $S_{0}$ has been characterized in Proposition 4.3.

Secondly, set the Lyapunov function $$\begin{array}{ll} V(\phi _{t}(q_{0}))=V(x, y, z) =&ac(c-2a)b_{4}(y - x)^{2}+a(c-2a)b_{4}^{2}(-cz+x^{2})^{2} + \frac{1}{2} [-b_{4}(c-2a)x^{2} + cb_{1}]^{2}. \end{array} $$

Proceeding as in^14,37,38,42,43,47,48,50–60^, one formulates the following result.

### Proposition 5.1

When $c - 2a > 0$, $0< b_{1} < a$, $b_{4} > 0$, $b_{2} = -cb_{4}$ and $b_{3} = -(c - 2a)b_{4}$, one derives the following assertions.

(i) If there exist $t_{1}$ and $t_{2}$ such that $t_{1} < t_{2}$ and $V(\phi _{t_{1}}(q_{0})) = V(\phi _{t_{2}}(q_{0}))$, then $q_{0}$ is one of equilibria of system (2).
(ii) If $\phi _{t}(q_{0})\rightarrow S_{0}$ as $t\rightarrow -\infty$, and $x(t; x_{0}) > 0$ for some $t\in \mathbb {R}$, then $V(S_{0}) > V(\phi _{t}(q_{0}))$ and $x(t; x_{0}) > 0$ for all $t\in \mathbb {R}$. Consequently, $q_{0}\in \gamma ^{+}$.

### Proof

i) For $c - 2a > 0$, $0< b_{1} < a$, $b_{4} > 0$, $b_{2} = -cb_{4}$ and $b_{3} = -(c - 2a)b_{4}$, one can compute the derivative of $V(\phi _{t}(q_{0}))$ along the solution $\phi _{t}(q_{0})$:

13 $$\begin{aligned} \begin{array}{lll} \frac{dV(\phi _{t}(q_{0}))}{dt}\big |_{(2)}= & {} 2ac(c-2a)b_{4}[(b_{1}-a)(y - x)^{2}-b_{4}(-cz+x^{2})^{2}] \le 0. \end{array} \end{aligned}$$

So, for all $t\in (t_{1}, t_{2})$, the condition (i) implies

14 $$\begin{aligned} y(t; y_{0}) - x(t; x_{0})\equiv -cz(t; z_{0})+x^{2}(t; x_{0})\equiv 0. \end{aligned}$$

In virtue of system (2), $\dot{x}(t; x_{0}) = a(y - x) = 0$ suggests $x(t) = x_{0}$ and $\dot{y}(t; y_{0}) = 0$, $\forall t \in \mathbb {R}$. $-cz+x^{2}=0$ implies $-cz+y^{2}=0$ for all $t \in \mathbb {R}$, i.e. $\dot{z}(t; z_{0}) = 0$. In a word, $q_{0}$ is one of equilibria, i.e.

15 $$\begin{aligned} \dot{x}(t; x_{0})\equiv \dot{y}(t; y_{0})\equiv \dot{z}(t; z_{0})\equiv 0. \end{aligned}$$

ii) Firstly, one proves $V(S_{0}) > V(\phi _{t}(q_{0}))$, $\forall t \in \mathbb {R}$. Otherwise, suppose $0 < V(S_{0}) \le V(\phi _{t_{0}}(q_{0}))$ for at least a $t_{0}\in \mathbb {R}$. This also yields that $q_{0}$ is one of equilibria of system (2), which leads to $q_{0} = S_{0}$ and $x(t; x_{0})=0$, $\forall t \in \mathbb {R}$ according to $\lim _{t\rightarrow -\infty }\phi _{t}(q_{0}) = S_{0}$. A contradiction occurs! Therefore, $V(S_{0}) > V(\phi _{t}(q_{0}))$, for all $t \in \mathbb {R}$.

Next, one proves $x(t; x_{0}) > 0$, $\forall t\in \mathbb {R}$. Assume by contrary that $x(t^{'}; x_{0}) \le 0$ for some $t^{'} \in \mathbb {R}$. From the hypothesis of (ii), there exists a $\tau \in \mathbb {R}$ such that $x(\tau , x_{0}) = 0$. Since $V(S_{0}) > V(\phi _{t}(q_{0}))$, $\forall t \in \mathbb {R}$, one has $\phi _{\tau }(q_{0})\in \{(x, y, z): V(S_{0}) > V(x, y, z)\} \cap \{(0, y, z)\}=\{(x, y, z): ac(c-2a)b_{4}y^{2}+a(c-2a)b_{4}^{2}c^{2}z^{2} + \frac{1}{2}c^{2}b_{1}^{2}<\frac{1}{2}c^{2}b_{1}^{2}\}=\varnothing$, which is a contradiction. Hence, it follows that $x(t; x_{0}) > 0$, $\forall t \in \mathbb {R}$. The proof of the proposition is finished. $\square$

Based on Proposition 5.1, the existence of heteroclinic orbits to $S_{0}$ and $S_{\pm }$ is derived in the following statement.

### Proposition 5.2

Consider $c - 2a > 0$, $0< b_{1} < a$, $b_{4} > 0$, $b_{2} = -cb_{4}$ and $b_{3} = -(c - 2a)b_{4}$. One has the statements as follows.

- Neither homoclinic orbits nor heteroclinic orbits to $S_{+}$ and $S_{-}$ exist in system (2).
- System (2) has a pair of symmetrical heteroclinic orbits to $S_{0}$ and $S_{\pm }$.

### Proof

a) Firstly, one proves that neither heteroclinic orbits nor homoclinic orbits to $S_{+}$ and $S_{-}$ exist in system (2). Assume by contrary that $\phi _{t}(q_{0})$ is a heteroclinic orbit or a homoclinic orbit to $S_{+}$ and $S_{-}$, i.e.

$$\begin{aligned} \lim _{t \rightarrow -\infty }\phi _{t}(q_{0}) = s_{-}, \lim _{t \rightarrow \infty }\phi _{t}(q_{0}) = s_{+}, \end{aligned}$$

where $s_{-}$ and $s_{+}$ satisfy either $\{s_{-}, s_{+}\}=\{S_{-},S_{+}\}$ or $s_{-}=s_{+}\in \{S_{0}, S_{-}, S_{+}\}$. From Eq. (13), one arrives at $V(s_{-}) \ge V(\phi _{t}(q_{0})) \ge V(s_{+})$ and thus $V(s_{-}) = V(s_{+})$ in either case, which also yields $V(\phi _{t}(q_{0}))= V(s_{+})$. According to Proposition 5.1(i), $q_{0}$ is one of equilibria. Therefore, system (2) has neither heteroclinic orbits nor homoclinic orbits joining $S_{+}$ and $S_{-}$.

b) Next, one proves that there exists a single heteroclinic orbit joining $S_{0}$ and $S_{+}$: $\gamma ^{+}(t)$. As $t \rightarrow \infty$, it follows Proposition 4.2 that $\phi _{t}(q_{0})$ approaches neither $S_{0}$ nor $S_{-}$. Hence, $\lim _{t \rightarrow \infty }\phi _{t}(q_{0}) = S_{+}$.

Finally, let us show that if system (2) has a second heteroclinic orbit to $S_{0}$ and $S_{+}$, then it coincides with $\gamma ^{+}$.

Suppose $\phi _{t}^{1}(q_{0})$ is a solution of system (2) that

$$\begin{aligned} \lim _{t \rightarrow -\infty }\phi _{t}^{1}(q_{0}) = s_{-}^{1}, \lim _{t \rightarrow \infty }\phi _{t}^{1}(q_{0}) = s_{+}^{1}, \end{aligned}$$

where $s_{-}^{1}$ and $s_{+}^{1}$ satisfy $\{s_{-}^{1}, s_{+}^{1}\}=\{S_{0},S_{+}\}$. Since *V* is decreasing, one has $V(s_{-}^{1}) \ge V(\phi _{t}^{1}(q_{0})) \ge V(s_{+}^{1})$ and $V(S_{0}) > V(S_{+})$. Therefore, one obtains $s_{-}^{1}=S_{0}$ and $s_{+}^{1}=S_{+}$, i.e.,

$$\begin{aligned} \lim _{t \rightarrow -\infty }\phi _{t}^{1}(q_{0}) = S_{0}, \lim _{t \rightarrow \infty }\phi _{t}^{1}(q_{0}) = S_{+}. \end{aligned}$$

It follows from Proposition 5.1(ii) that $\phi _{t}^{1}(q_{0}) = \gamma ^{+}$.

**Figure 10:**
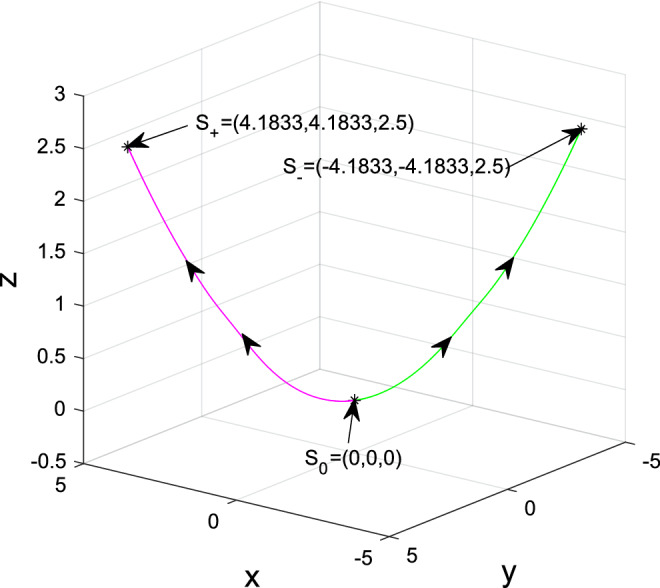
Heteroclinic orbits to $S_{0}$ and $S_{\pm }$ of system (2) for $(a,b_{1},b_{2},b_{3},b_{4},c)=(3,2.5, -7, -1,1,7)$ and initial values $(x_{0}^{',''}, y_{0}^{',''}, z_{0}^{',''})=(\pm 1.382\times 1e^{-6}, \pm \frac{5.5}{3}\times 1.382\times 1e^{-6}, \frac{6.05}{9}\times 1.382\times 1.382\times 1e^{-12})$ on the unstable manifolds of $S_{0}$, i.e., the $W^{u}_{loc}(S_{0})$ characterized in Proposition 4.3.

Since system (2) is symmetrical with respect to the *z*-axis, $\gamma ^{-}$ is another unique heteroclinic orbit to $S_{0}$ and $S_{-}$. Figure 10 verifies the correctness of the theoretical result. Thus proof is completed. $\square$

### Remark 5.3

It follows from Proposition 5.2 that the special case of system (2), i.e., the one (1) has not heteroclinic orbits to $S_{0}$ and $S_{\pm }$ for $b_{2} = b_{4} = 0$. How to prove the existence of heteroclinic orbits of system (1) will be our future work, if they exist. Compared with other Lorenz-like systems^14,37,38,42,43,47,48,50–60^, it is more difficult to construct the Lyapunov function for system (2) with more nonlinear terms.

## Conclusions

This note reports a new 3D cubic Lorenz-like system, which contains the existing one as special cases and generates rich dynamics, such as generic and degenerate pitchfork bifurcation, Hopf bifurcation, infinitely many singularly degenerate heteroclinic cycles with nearby Chen-like attractors, etc. Using Lyapunov functions, we prove that the parabolic type equilibria are globally exponentially asymptotically stable, and there exists a pair of heteroclinic orbits to the origin and two symmetrical equilibria.

In future work, other important dynamics of that system, such as homoclinic orbit, invariant algebraic surface, positively invariant set, the forming mechanism of chaotic attractor and so on, require further analytical descriptions to complete its mathematical treatment. We also hope that the basic ideas and the self-contained approach presented in this paper can be applied to explore other similar chaotic/hyperchaotic systems, i.e.,

16 $$\begin{aligned} \left\{ \begin{array}{ll} \dot{x}&{}=a(y - x),\\ \dot{y}&{}=b_{1}y+b_{2}yz+b_{3}xz+b_{4}x^{2}y+p_{1}w, \\ \dot{z}&{}= -cz + y^{2},\\ \dot{w}&{}= q_{1}w + q_{2}y, \end{array}\right. \end{aligned}$$

17 $$\begin{aligned} \left\{ \begin{array}{ll} \dot{x}&{}=a(y - x),\\ \dot{y}&{}=b_{2}yz+b_{3}xz+b_{4}x^{2}y+p_{1}w, \\ \dot{z}&{}= -cz + y^{2},\\ \dot{w}&{}= q_{1}w + q_{3}x, \end{array}\right. \end{aligned}$$

18 $$\begin{aligned} \left\{ \begin{array}{ll} \dot{x}&{}=a(y - x),\\ \dot{y}&{}=b_{1}y+b_{2}yz+b_{3}xz+b_{4}x^{2}y+p_{1}w+p_{2}x, \\ \dot{z}&{}= -cz + y^{2}+xy,\\ \dot{w}&{}= q_{1}w + q_{2}y + q_{3}x, \end{array}\right. \end{aligned}$$

where $a \ne 0$, $c, b_{i}, p_{1}, p_{1}, p_{2}, q_{1}, q_{2}, q_{3} \in \mathbb {R}$, $i=1, 2, 3, 4$, etc. Preliminary studies show that there might exist chaotic/hyperchaotic attractors, generic and degenerate pitchfork bifurcation, Hopf bifurcation, singularly degenerate heteroclinic cycles, globally exponentially asymptotically stable parabolic type equilibria, a pair of heteroclinic orbits to the origin and two symmetrical equilibria in system (16–18). We also guess that the dynamics also exist in higher dimensional analogues.

## Data Availability

All data generated or analyzed during this study are included in this published article.
